# Custom foot orthoses improve first-step pain in individuals with unilateral plantar fasciopathy: a pragmatic randomised controlled trial

**DOI:** 10.1186/s12891-018-2131-6

**Published:** 2018-07-18

**Authors:** Chris Bishop, Dominic Thewlis, Susan Hillier

**Affiliations:** 1 Alliance for Research in Exercise, Nutrition and Activity (ARENA), University of South Australia, Adelaide, East Campus, North Terrace, SA 5000 Australia; 2 Sansom Institute for Health Research, University of South Australia, Adelaide, Australia; 3 Centre for Orthopaedic and Trauma Research, University of Adelaide, Adelaide, Australia

**Keywords:** Plantar fascia, Foot orthoses, Pain, Footwear, Ultrasound

## Abstract

**Background:**

Foot orthoses are routinely used to treat plantar fasciopathy in clinical practice. However, minimal evidence exists as to the effect of both truly custom designed foot orthoses, as well as that of the shoe the foot orthoses are placed into. This study investigated the effect of wearing custom foot orthoses and new athletic footwear on first-step pain, average 24-h pain and plantar fascia thickness in people with unilateral plantar fasciopathy over 12 weeks.

**Methods:**

A parallel, three-arm randomised controlled trial with blinding of participants and assessors. 60 participants diagnosed with unilateral plantar fasciopathy were randomised to either custom foot orthoses and new shoes (orthoses group), a sham insole with a new shoes (shoe group) or a sham insole placed in the participant’s regular shoes (control group). Primary outcome was first-step pain. Secondary outcomes were average 24-h pain and plantar fascia thickness measured on ultrasound. Outcomes were assessed at baseline, 4 week and 12 week trial time-points.

**Results:**

At 4 weeks, the orthoses group reported less first-step pain (*p* = 0.002) compared to the control group. At 12 weeks, the orthoses group reported less first-step pain compared to both the shoe (*p* = < 0.001) and sham (*p* = 0.01) groups. Both the orthoses (*p* = < 0.001) and shoe (*p* = 0.006) groups reported less average 24-h pain compared to the control group at 4 and 12 weeks. The orthoses group demonstrated reduced plantar fascia thickness on ultrasound compared to both the shoe (*p* = 0.032) and control groups (*p* = 0.011).

**Conclusions:**

Custom foot orthoses in new shoes improve first-step pain and reduce plantar fascia thickness over a period of 12 weeks compared to new shoes alone or a sham intervention.

**Trial registration:**

Australian New Zealand Clinical Trials Registry (ACTRN 12613000446763). Submitted on the 10th of April 2013 and registered on the 18th of April 2013.

**Electronic supplementary material:**

The online version of this article (10.1186/s12891-018-2131-6) contains supplementary material, which is available to authorized users.

## Background

Plantar heel pain is a common clinical presentation of the foot, estimated to affect one in ten people over their lifetime [1] with an indirect cost to the United States healthcare system of $390 million per year [2]. The greater significance of the problem relates to the burden placed on the individual; the presence of pain in the plantar heel affects foot-related quality of life [3–5] and alters the way people walk [6–8]. Therefore, to effectively manage plantar heel pain, it is important that treatments are optimised to reduce its burden.

Pain in the plantar heel can be managed by a range of treatments, with foot orthoses being one commonly used by health professionals [3]. Treatment using foot orthoses has been shown to be effective in previous clinical trials [9–14], although the mechanism by which foot orthoses exert their effect is not clear. However, two recent systematic reviews have presented contradictory results regarding the effect of foot orthoses on pain [15, 16]. Whittaker et al. concluded that the use of foot orthoses in individuals with plantar heel pain is beneficial in the medium term (7 to 12 weeks) 12 weeks regardless of whether prefabricated, accommodative or custom orthoses are used [15]. In contrast, Rasenberg et al. concluded that foot orthoses are not superior for improving pain or function compared to sham or other conservative treatments using largely the same body of evidence [16]. The later conclusion was based on small, yet non-significant effects favouring foot orthoses. On face value, these contradictory results present a high-degree of confusion and uncertainty for clinicians as to the effect of foot orthoses, and how to appropriately use them, in the treatment of plantar heel pain. However, a recent editorial has identified that the difference in the conclusions between the two reviews is a result of the different pain outcome extracted by the respective authors from one of the studies included in the reviews [17]. This suggests that current pooled evidence demonstrates a small effect favouring foot orthoses, with the need for further high-quality randomised controlled trials to interpret whether such an effect is important in the context of clinical management.

From a clinical practice standpoint, whilst custom devices are most commonly prescribed in practice [18], the literature supports prefabricated orthoses as being just as beneficial as custom devices in the treatment of plantar heel pain [15]. Given the documented effect of prefabricated orthoses, the question is not a matter of choice between custom or prefabricated design, but rather which orthoses design has the ability to manipulate the kinematics and kinetics of the foot to unload the plantar fascia. Although a recent review suggests that health practitioners may consider using prefabricated orthosess that are appropriately contoured to the foot [15], the hypothesis with the use of custom foot orthoses is the ability to use custom geometry to exert individual reaction forces at the level of an individual joint(s) of the foot that may not necessarily be possible with the use of prefabricated orthoses. We acknowledge that the research is inconclusive as to whether custom foot orthoses are more effective than prefabricated or accommodative devices. Some research shows non-significant and small effects on pain with the use of custom orthoses [9–12], yet other studies show clear improvement in pain [13, 14]. This intervention variability is likely due to the methodological biases that exist within individual studies in regards to the prescription design that limit the transfer of findings into clinical practice [15].

To better understand the effect of foot orthoses on plantar heel pain, there is a need for a pragmatic trial that best represents standard care in clinical practice. Recent evidence suggests a wide variety of prescriptions used to manufacture custom foot orthoses in practice [19]. We feel improved translation of orthoses research findings into practice relates to three key considerations. Firstly, a recent criticism of the foot orthoses literature was that most clinical studies standardise the type of prescription approach across all participants [20]. By virtue of their location between the foot and shoe, foot orthoses have the ability to alter the biomechanics of the foot and the forces acting on the plantar heel [21–24]. Using the same orthoses prescription for all individuals does not necessarily allow the design of orthoses to reflect the requirements of each individual in terms of required biomechanical effect, material stiffness and device comfort in order to reduce pain and/or improve function. Secondly, previous foot orthoses research has assumed any effect of intervention is purely a result of the foot orthoses, even though footwear has been shown to affect foot and ankle function [25]. Although it is conceivable that the shoes the orthoses are worn in may, in part, contribute to the success (or lack of success) of orthoses therapy, this has not been controlled for in previous trials. Ignoring any potential effect of footwear may then incorrectly attribute the entire effect of the shoe-orthoses combination to the orthoses itself. Thirdly, an accurate diagnosis of pathology is required to prescribe the most appropriate treatment; while the terminology used to describe pain in the plantar heel has improved with the adoption of plantar heel pain [26], it still is not a specific diagnosis of pathology. Pain in the plantar heel can be caused by bone, muscle, fascia and neural structure pathology within the plantar heel region [27–29], and it is likely that each structures requirements from and/or response to treatment will be different. Based on a tissue stress approach to treatment [30], it is important that the specific site of pathology is identified in order to design an intervention to move the stresses away from the pathological tissue.

The mixed quality of the work in this field makes it difficult to conclude on the effectiveness of custom foot orthoses for the treatment of pathology in the plantar heel. Factors such as not concealing treatment allocation, not blinding assessors, not diagnosing the actual pathology, not designing orthoses to suit the needs of the individual and not including true control groups all limit the ability to translate research findings into practice [31]. Without addressing such limitations, it will not be possible to guide clinicians about the appropriateness of using custom foot orthoses as a treatment option.

## Methods

### Trial aims

The primary aim of this clinical trial was to investigate the effect of custom foot orthoses in reducing self-reported first-step pain over 12 weeks compared to the shoes they were placed in and a sham treatment. Based on previous results of foot orthoses, we hypothesised that the orthoses group would report less pain at 12 weeks compared to a sham group. Our secondary aims were to explore the effect of custom foot orthoses on both average 24-h pain and plantar fascia thickness over 12 weeks.

### Study design

A parallel, three-arm randomised controlled trial (RCT) with concealed allocation and blinding of participants and assessors was conducted. Data were collected at three time points: baseline (intervention allocation); four weeks from baseline (4 weeks into the intervention); and 12 weeks from baseline (12 weeks, end of intervention). 12 weeks from baseline was the end-point and main assessment of the trial. The protocol for this study was approved by the local Human Research Ethics Committee and research standards adhered to the World Medical Association’s Declaration of Helsinki [32] and the 2010 CONSORT Statement [33]. All participants provided written informed consent. The protocol was registered on the Australian New Zealand Clinical Trials Registry **(**ACTRN 12613000446763 registered on the 18th of April 2013**).**

To enhance the trial, a number of minor modifications were made to the original registered protocol; A) the unmodified control shoe had a thin cambrelle liner applied to the innersole to be the same presentation as the other conditions, B) a daily pain diary was used instead of a weekly one to provide a more consistent measure of pain, C) an increase of sample size from 51 to 60 was made based on the advice of a new statistician and analytical approach as highlighted in the update to protocol and D) the 52-week from baseline timeline was also dropped due to issues with retention of participants.

### Sample size

An *a prior* sample size calculation was performed based on a minimal important difference in first-step pain of 19 mm on a 100 mm Visual analogue scale (VAS) (effect size Cohen’s *d* = 0.8) [34]. Sixty participants (20 participants, three groups) were required to detect an effect size of 0.8 comparing any two groups, using alpha = 0.025 to take account of multiple comparisons and assuming 0.6 correlation over time. This was linear mixed effects model with Time, Group and Time-Group interaction.

### Recruitment and eligibility criteria

The trial was conducted between April 2013 and December 2014 (trial duration was 21 months). Recruitment was via expressions of interest from local advertising. Trial eligibility criteria is outlined in Table 1. Three methods were used to diagnose plantar fasciopathy. Clinically, pain was first required to be reproduced with manual palpation of the medial tubercle where the plantar fascia attaches to the calcaneus. The minimum threshold of pain established of ≥20 mm on a 100 mm VAS ensured a minimal important difference could be obtained [34], if the effect were to be true. Pain of neural origin was then excluded based on clinical tests, with any individual reporting reproduction of symptoms with dorsiflexion/eversion or plantarflexion/inversion nerve compression tests excluded [27]. Finally, a diagnosis of plantar fasciopathy was confirmed on ultrasound (IU22, Phillips, Netherlands) by the presence of (at least one) diffuse or localised hypoechoic areas within a thickened calcaneal attachment (i.e. ≥ 4.0 mm), evidence of biconvexity, collection of fluid around the fascia or intra-fascial calcification [35–37]. Ultrasounds were taken by investigator (CB) whom is a qualified podiatrist with 10 years’ experience in the diagnosis and management of plantar heel pain. This investigator was trained by a musculoskeletal sonographer with 20 years imaging experience and demonstrated excellent reliability relative to a trained musculoskeletal sonographer in pre-trial testing (intra-session intra-class correlation coefficient (ICC) = 0.949 [95% CI = 0.892–0.976], intra-day ICC = 0.861 [95% Confidence interval (CI) = 0.708–0.934] and inter-day ICC = 0.837 [95% CI = 0.658–0.923]).

**Table 1:** Trial eligibility criteria

Inclusion criteria	Exclusion criteria
18–60 years of age Pain on both self-report AND palpation of the medical calcaneal tubercle as ≥20 mm on a 100 mm VAS [34] Duration of symptoms ≥ four weeks Diagnosis of plantar fasciopathy on ultrasound as proximal attachment of plantar fascia to calcaneus measuring ≥4.0 mm. [37]	Current or previous use of foot orthoses (prefabricated or custom) Had received treatment for current symptoms Had purchased new footwear in the last four weeks Bilateral symptoms Neural symptoms and/or reproduction of pain with neural testing Corticosteroid injection in the heel in the last six months Pregnancy Medical history of Diabetes (Type I or II), inflammatory arthropathies, or neuromuscular conditions Previous lower limb orthopaedic surgery

If a volunteer met all the eligibility criteria, they provided written informed consent and were enrolled in the trial. Anthropometric data were recorded to define stature. Baseline trial characteristics were defined using 100 mm VAS (to define first-step and average 24-h pain) and ultrasound used to measure plantar fascia thickness (Phillips IU22, Phillips, Japan). A non-weight bearing plaster cast was taken of all participants’ feet and they were told the casts would be used to manufacture the intervention. A biomechanical examination was then performed on all participants in order to obtain the required data for an orthoses prescription. Participants also provided a pair of shoes that they were willing to wear exclusively for the duration of the trial. This was in the event they were allocated to the control group. Any medication taken by participants throughout the trial was documented and then monitored on a weekly basis. Investigator (CB) screened all volunteers, performed all eligibility screening, conducted the biomechanical analysis, casted for and prescribed all foot orthoses.

### Interventions

Participants were randomly allocated to one of three groups: 1) the control group received a sham intervention which consisted of their existing footwear and a sham insole made from 0.7 mm non-textured cambrelle; 2) the shoe group acted as a positive-control group and received new athletic shoes (ASICS Nimbus 14, ASICS Corp. Japan); and 3) the orthoses group received custom foot orthoses inserted into new athletic footwear (ASICS Nimbus 14, ASICS Corp. Japan). Based on the recommendations of Lee et al. [31], the sham insole was also used as the insole in the new shoes and the top cover of the orthoses. All footwear were fitted by Investigator (CB) using a men’s and women’s adult Brannock device (The Brannock Device Co., USA). The foot orthoses used in this trial were customised to the foot of the individual and represented both the most common prescription habits by podiatrists [18], as well as the results of a recent Delphi consensus [20]. The orthoses prescription for each participant is outlined in Table 2. Additional file 1 provides technical footwear specifications of the shoe prescribed as well as the guidelines for the manufacture of orthoses devices. All orthoses were manufactured from 4.0 mm polypropylene and had a 350 kg/m^3^ density heel post to stabilise the rearfoot. All orthoses were made by investigator (CB) who has 10 years’ experience in the manufacture of custom foot orthoses in practice. Participants were told they could not wear other shoes whilst participating in the trial. To monitor compliance to protocol, participants recorded the time spent wearing the intervention each day in a diary. The amount each participant wore their allocated intervention was referenced to previous benchmarks or normal weight-bearing activity defined in a use of time database of 3276 adults aged 18–95 years held by the Alliance for Research in Exercise, Nutrition and Activity (ARENA) at the University of South Australia (Table 3). In absence of a defined criteria of time needed to achieve a therapeutic response, benchmarking the amount of time each participant wore their intervention relative to expected normal activity of the population gives context to the relative dose-response of the intervention and shows our participants were active and not sedentary.

**Table 2:** Participant specific orthoses prescription variables **–** Orthoses prescription variables

Subject No.	Prescription Variable							Material Variable		
	Poured to neutral	Forefoot balanced to rearfoot	MLA height of plaster (mm)	Medial skive	Lateral expansion	1st Met cut out	PF Accom	4 mm poly	Heel post	Top cover
1	√	√	38	x	√	x	x	√	√	√
2	√	√	36	x	√	x	x	√	√	√
3	√	√	43	x	√	x	x	√	√	√
4	√	√	38	x	√	x	x	√	√	√
5	√	√	41	x	√	x	x	√	√	√
6	√	√	26	x	√	x	x	√	√	√
7	√	√	30	x	√	x	x	√	√	√
8	√	√	26	x	√	x	x	√	√	√
9	√	√	44	x	√	x	x	√	√	√
10	√	√	36	x	√	x	x	√	√	√
11	√	√	34	x	√	x	x	√	√	√
12	√	√	28	x	√	x	x	√	√	√
13	√	√	36	x	√	x	x	√	√	√
14	√	√	30	x	√	x	x	√	√	√
15	√	√	40	x	√	x	x	√	√	√
16	√	√	48	x	√	x	x	√	√	√
17	√	√	28	x	√	x	x	√	√	√
18	√	√	44	x	√	x	x	√	√	√
19	√	√	35	x	√	x	x	√	√	√
20	√	√	43	x	√	x	x	√	√	√

**Table 3:** Benchmark data for normal activity patterns of male and female adults

Age Bracket	Males		Females	
	Hours/day	Hours/trial	Hours/day	Hours/trial
20–29 years	5.85	491.40	5.47	459.20
30–39 years	4.85	407.40	5.93	498.40
40–49 years	5.65	474.60	6.32	530.60
50–59 years	5.27	442.40	5.50	462.00

### Randomisation, treatment allocation and blinding

Group allocation was conducted via a researcher blind to recruitment using a computer generated block (4 × 15 blocks) random number sequence, after the initial assessment outlined above. Participants were blinded as to the exact nature of the trial, and simply told that the trial was investigating the effect of three different insoles in treating plantar heel pain. A blinded assessor was used to process all outcome data.

### Research outcomes

First-step pain was the primary clinical outcome of interest as it is commonly reported by patients in clinical practice [38]. We defined first-step pain as the pain experienced in the plantar heel when putting the foot on the ground to get up after a period of extended rest**.** First-step pain was assessed using a horizontal 100 mm VAS at the baseline, 4 week and 12 week trial time points. Two secondary outcomes were also assessed at each trial time point; average 24-h pain and plantar fascia thickness. We defined average 24-h pain as the average pain experienced in the plantar heel over the previous 24-h period. This was assessed on a horizontal 100 mm VAS. The dorso-plantar thickness of the plantar fascia was measured (mm) by ultrasound at the point where the fascia crosses the anterior aspect of the inferior calcaneal border [37]. Biomechanical outcomes were also captured (as detailed in the trial registry) and will be presented in a follow-up article.

### Data analysis

Intention to treat analysis was used to compare groups across trial time-points. A baseline observation carried forward approach (rather than last observation) was used as this has been shown to provide a more conservative estimate of treatment effect [39, 40]. Data were assessed for normality using Shapiro-Wilks tests (*p* = 0.05). A mixed model, with group designated as fixed effects and individual subjects as random effects was used to analyse the differences between groups at each trial time-point. Baseline outcome data was used as a co-variate. Post-hoc Holm-Bonferroni corrections were used to account for multiple comparisons. Cohen’s *d* was calculated to demonstrate the size of effect present and interpreted relative to thresholds from the literature [41]. A change of ≥19 mm on a 100 mm VAS [34] was deemed an important clinical change.

## Results

**Fig. 1:**
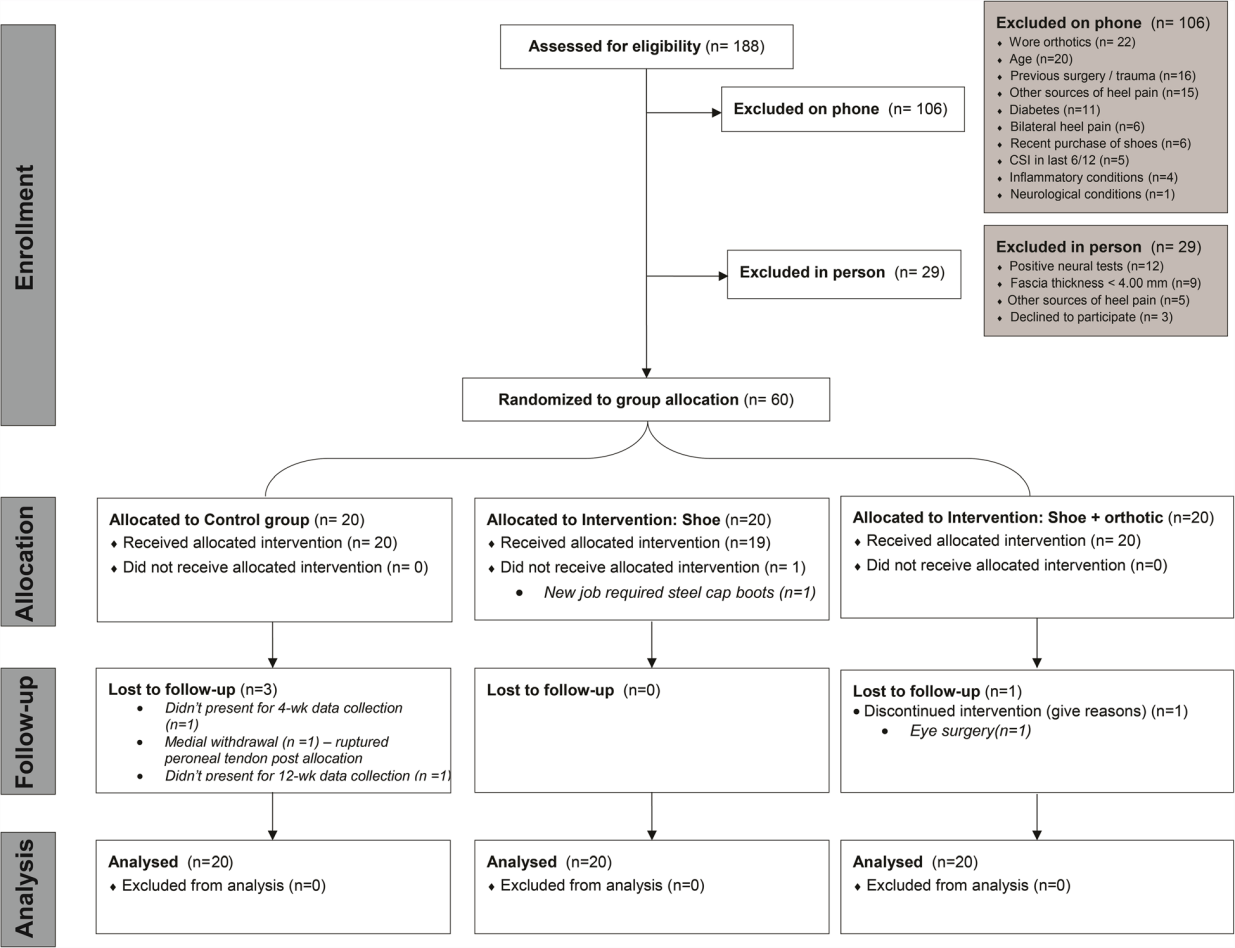
CONSORT flowchart - the recruitment process of participants into the trial

**Fig. 2:**
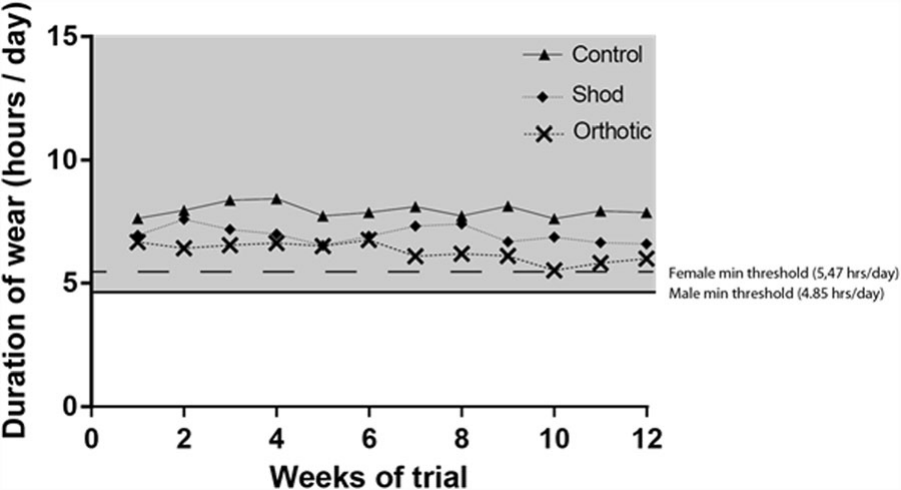
Duration of intervention wear throughout the trial. The minimum required threshold for males (bold line) and females (broken line) are plotted. The grey shaded area signifies the zone of acceptable intervention wear per day

**Fig. 3:**
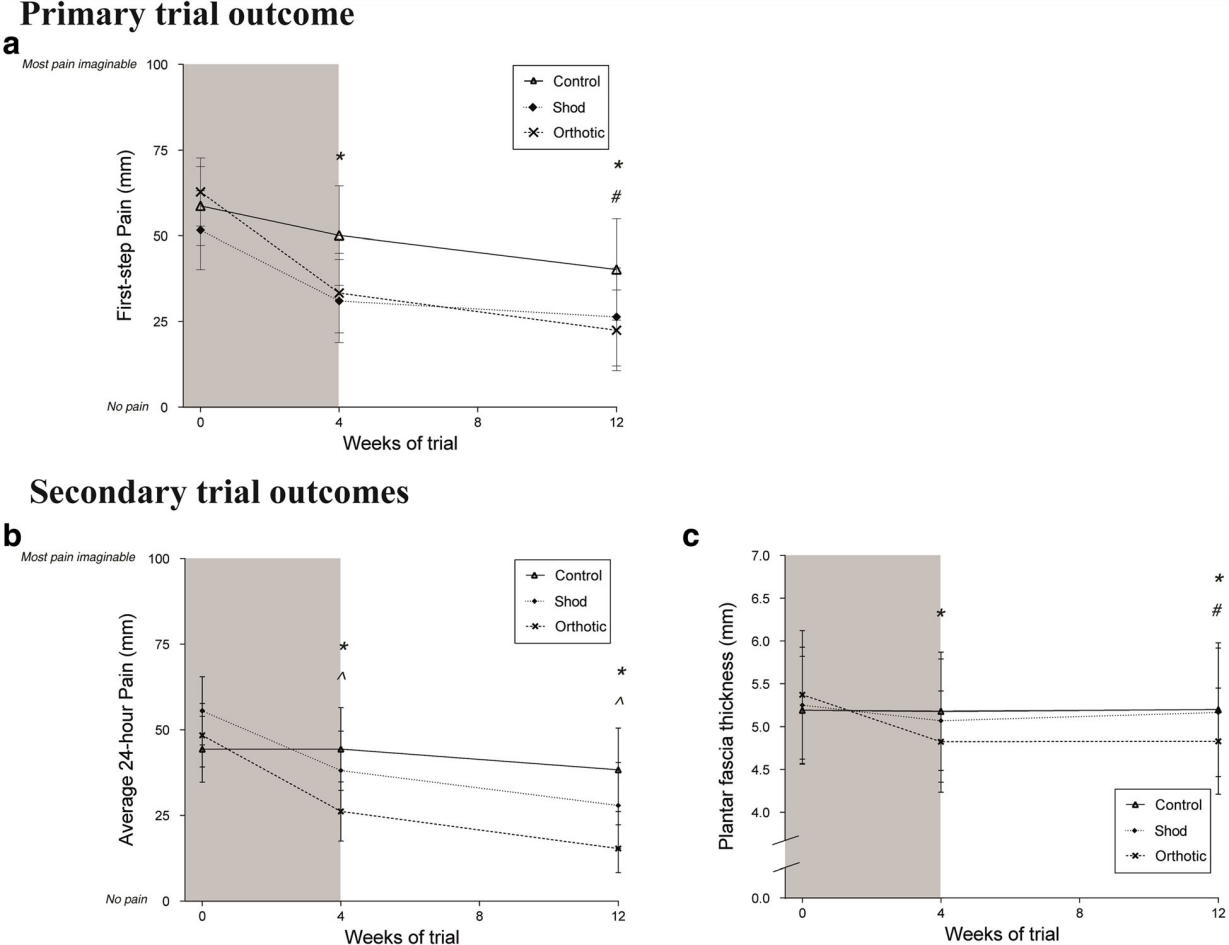
Between group comparison main effects of trial outcome measures. **a** First-step pain, **b** average 24-h pain, **c** Plantar fascia thickness

A CONSORT flowchart is presented in Fig. 1 to demonstrate the recruitment, allocation and flow of the trial. Sixty participants were randomly allocated to one of three groups. Four participants were lost to the trial for reasons outlined in Fig. 1. Baseline group characteristics are provided in Table 4. Mean compliance of wearing the intervention exceeded the defined thresholds for males and females in all groups (Fig. 2). There were no significant differences identified between groups for daily intervention wear time (*p* = 0.491). Main effects of condition for all trial outcomes are provided in Fig. 3. Only significant post-hoc comparisons are reported in text.

**Table 4:** Baseline characteristics of participants with plantar fasciopathy allocated to the control, shoe or orthoses groups

Variable		Control group		Shoe group		Orthoses group		*p*-value
		13 F:7 M		12F: 8 M		14F: 6 M		
		Mean	SD	Mean	SD	Mean	SD	
Age *(years)*		44.7	13.3	44.9	14.5	44.5	13.0	0.996
Duration of symptoms *(month)*		6.0	3.1	6.1	3.3	6.2	2.5	0.987
Baseline pain (100 mm VAS)	First-step	58.7	24.6	51.7	24.6	62.8	21.3	0.324
	Average 24-h	44.4	20.6	55.6	21.3	48.4	19.8	0.227
Height *(m)*		1.67	0.08	1.69	0.08	1.70	0.09	0.635
Body mass *(kg)*		76.1	23.4	85.4	25.6	80.1	18.8	0.725
BMI *(kg/m*^*2*^*)*		27.1	8.3	29.8	9.3	27.7	5.6	0.681
Foot length *(mm)*		247.2	12.8	246.5	16.7	244.7	22.3	0.900
		246.1	13.3	247.0	17.0	245.3	22.6	0.989
Navicular height *(mm)*	*Symp*	35.2	4.9	38.6	6.9	36.2	6.6	0.292
	*Non-symp*	35.8	5.5	38.8	6.3	36.4	6.9	0.363
Normalised navicular height truncated (NNHt)	*Symp*	0.20	0.03	0.22	0.04	0.20	0.05	0.290
	*Non-symp*	0.20	0.03	0.22	0.05	0.21	0.04	0.400

*Abbreviations*: *mnth* months, *VAS* visual analogue scale, *m* metres, *kg* kilograms, *BMI* body mass index, *mm* millimetres, *Symp* the symptomatic foot, *Non-symp* the non-symptomatic foot, *F* female participants, *M* male participants, *Mean* population mean, *SD* standard deviation of the mean. Statistical significance was set at 0.05

### Primary outcome

The fixed effects model indicated a significant main effect of group (*p* = 0.015) and time (*p* = < 0.001) for first-step pain. The model did not detect a significant group × time interaction (*p* = 0.255). Over the first four weeks of the trial, there was a significant improvement in first-step pain in the orthoses group compared to the control group (Mean difference (MD) = 20.9 [9.5–34.0] mm, *p* = 0.002, *d* = 0.820). At 12 weeks, the orthoses group reported lower first-step pain compared to both the shoe group (MD = 17.4 [7.8–37.4] mm, *p* = < 0.001, *d* = 0.394) and control group (MD = 24.3 [4.3–30.5] mm, *p* = 0.01, *d* = 0.675). No significant effects of group were identified between the 4 week and 12 week trial time points.

### Secondary outcomes

#### Average 24-h pain

The fixed effects model indicated a significant main effect of group (*p* = 0.04) and time (*p* = < 0.001) for average 24-h pain. The model detected a significant group × time interaction (*p* = 0.049). At 4 weeks, both the orthoses group (MD = 22.3 [7.0–37.6] mm, *p* = 0.005, *d* = 0.647) and shoe group (MD = 17.6 [2.3–32.8] mm, *p* = 0.025, *d* = 0.511) reported lower average 24-h pain compared to the control group. At 12 weeks, both the orthoses (MD = 28.2 [12.9–43.4] mm, *p* = < 0.001, *d* = 0.821) and shoe groups (MD = 21.6 [6.3–36.9] mm, *p* = 0.006, *d* = 0.500) reported lower average 24-h pain compared to the control group. No significant effects of group were identified between the 4 week and 12 week trial time points.

#### Plantar fascia thickness

The fixed effects model indicated no effect of group (*p* = 0.354) but a significant main effect of time (*p* = 0.002) for plantar fascia thickness. The model detected a significant group × time interaction (*p* = < 0.001). Over the first four weeks of the trial, the orthoses group demonstrated a reduction in plantar fascia thickness compared to the control group (MD = 0.54 [0.12–0.95] mm, *p* = 0.012, *d* = 0.659). At 12 weeks, the orthoses group reported a reduction in plantar fascia thickness compared to both the control (MD = 0.55 [0.13–0.97] mm, *p* = 0.011, *d* = 0.546) and shoe groups (MD = 0.46 [0.04–0.88] mm, *p* = 0.032, *d* = 0.381).

## Discussion

### The effect of custom foot orthoses on pain associated with plantar fasciopathy

This RCT investigated the effect of custom foot orthoses in new shoes for the treatment of plantar fasciopathy over a period of 12 weeks. The primary aim of this trial was to investigate the effect of custom foot orthoses on first-step pain over 12 weeks. We hypothesised that the use of custom foot orthoses would significantly improve self-reported pain compared to sham treatment over 12 weeks. The results of the study support our primary hypothesis: participants who used custom foot orthoses reported less pain at 12 weeks than those participants prescribed a sham treatment. Where previous clinical trials have also identified benefits of wearing custom foot orthoses in the treatment of plantar heel pain [13, 14], the results of these trials have represented the combined effect of the orthoses and shoe. No previous research has isolated the independent effect of the orthoses when worn in shoes. In this RCT, given the effect of shoe was accounted for, the benefit of wearing custom foot orthoses was in their ability to reduce first-step pain at 12 weeks. The finding of improvement in first-step pain wearing orthoses is consistent with the findings of Lynch and colleagues [14] and Martin and colleagues [11] who reported changes of a similar magnitude (44 mm and 53 mm respectively, vs. 40 mm in our study on a 100 mm VAS). Our data continue to support the benefit of treating plantar fasciopathy with custom foot orthoses. It is important to acknowledge however that although we demonstrate clinical significance with our data, the issue of clinical relevance may or may not be resolved. Our confidence intervals for the mean difference are wide and some reach beyond the minimal clinical important difference. Given the analysis conducted in this trial focussed on the mean population, future analysis of this dataset may benefit from dichotomising the population into sub-groups of those who did and did not either improve based on self-reported outcomes.

The secondary aims of this trial was to investigate the effect of custom foot orthoses on average 24-h pain and plantar fascia thickness over a period of 12 weeks. In respect to average 24-h pain, both the orthoses and shoe groups improved at the same rate and reported less average-24 h pain than the control group at 12 weeks. This may indicate that the response to custom foot orthoses in people with plantar fasciopathy depends on the type of pain experienced. In the event of first-step pain, the use of custom foot orthoses is more effective than simply purchasing a new shoe or sham treatment. This is important in the context of commonly reported symptoms of patients with plantar heel pain whereby there is a potential solution to the struggle of taking that first step out of bed or up out of a chair after a long period of sitting. Where as in the event of average 24-h pain, the use of custom foot orthoses was no more effective than the use of a new shoe. This indicates that perhaps simply cushioning in a shoe is all that is required to assist average 24-h pain. However, we must acknowledge that trial participants were confused by this outcome and it required a lot of explaining. Further most participants reported that this is not a significant pain and debilitating for them, and that naturally feet are tired and sore at the end of the day. The outcome was included in this trial as an attempt to better understand the overall concept of pain. We did not survey patient’s pre-trial as to whether this outcome was important. In hindsight, it is possible that this outcome is redundant and not specific to the discussion of plantar heel pain. Future trials attempting to further explore specific types of pain may benefit from targeting outcomes that are important and debilitating to the individual.

### The effect of custom foot orthoses on plantar fascia thickness

A novel finding of this RCT was the effect of custom foot orthoses on the thickness of the plantar fascia at its attachment with the calcaneus. The physiological response of the plantar fascia to treatment over time has previously been investigated with the use of corticosteroids [40]. Our data suggest that custom foot orthoses have a similar effect (13.1% change vs. 10.1% change in our study) in reducing the thickness of the plantar fascia in people with plantar fasciopathy. This indicates that plantar fascia swelling identified on imaging may actually be reversible [40], and if proven so, may support a similar pathomechanical continuum model as seen in tendons [42]. It is important that future research is designed to explore the concept further.

### Trial limitations & implications for future research

The results of this RCT should be interpreted with respect to its limitations. It is unknown, based on our data, whether the effects of custom foot orthoses are sustained over a period of time longer than 12 weeks. Although our data are consistent with Whittaker’s findings of medium term effects [15], previous literature has shown that the benefits of foot orthoses plateau after three months compared to natural progression of symptoms [9]. Although the original intention of this trial was to collect outcome data at 52 weeks, patient retention and trial timelines made this not possible. Any attempt to further investigate the long-term effects of custom foot orthoses should not only consider the time frame of trial assessment points, but also the effect of cyclical loading on the stiffness of the orthoses material. It is foreseeable that with high frequency cyclic loading over time, there could be a change in the material properties of the orthoses that may influence its response to mechanical load [43]. Likewise we must acknowledge limitations relating to the experimental conditions defined in this study. Firstly, the use a subject-chosen control condition with thin insole may have potentially provided a therapeutic benefit and could potentially mask clinical benefits of orthoses [44]. Although we feel this is unlikely, and that there are clear benefits to the use of control conditions in orthoses research and that patient’s own footwear is actually recommended as the best control [45, 46], the interpretation of the results requires an assumption that the control intervention was mechanically inert. Secondly, we must recognise that the results from the shoe group may in fact be specific to the neutral shoe worn in this study. It is fair to state that similar to the argument of custom orthoses prescription, participants in this study may have benefited from individualised shoe prescription. This is a required area of future research.

Compliance to protocol was self-reported as a way to monitor fidelity that participants actively adhered to the research protocol and wore the intervention allocated. Future trials need to develop methods to assess whether the amount of time an intervention was worn is sufficient to receive a therapeutic benefit. In addition, we acknowledge the psychological effect of treatment and the influence this has on outcomes. Future trials may benefit from measuring expectancy of benefit and credibility of the intervention allocated as this is likely important in the early stages of treatment (particularly in the sham group) and shown to be insightful in previous orthoses related studies [47]. We also must consider that the inclusion of participants in this study relied heavily on a diagnosis of plantar fasciopathy on ultrasound. Although ultrasound findings were considered in addition to other clinic features that were required to be present in order to be deemed eligible, we acknowledge the methodological issues that exist in some studies and that further work is required to assist in establishing a better diagnostic framework and whether imaging is or is not required for purposes of diagnosis. Finally, it is important to acknowledge the variability in individual response, and this may have resulted from a combination of differences in orthoses design, differences in the response of the individual to the treatment condition provided or differences between male and female shoes. Although we acknowledge that we may have received the same response to treatment using a prefabricated device, we feel a truly customised approach to the prescription of orthoses is an important consideration in terms of translation of research findings to clinical practice, as it is unlikely that all individuals with plantar heel pain require the same intervention. However, in order to determine if an individualised custom orthoses is superior to a standard prescription or prefabricated device that another trial is required.

## Conclusion

Custom foot orthoses appear to be effective in the treatment of first-step pain in individuals with plantar fasciopathy over a period of 12 weeks. Compared to wearing new shoes alone or a sham treatment, using custom foot orthoses resulted in less first-step pain and a less thickened plantar fascia. Custom foot orthoses were no more effective than wearing new shoes in the reduction of average 24-h pain. Our results support the use of custom foot orthoses in clinical practice for the treatment of first-step pain in individuals diagnosed with plantar fasciopathy.

## Additional file

Additional file 1: Instructional. The guidelines for the manufacture and/or design of each intervention. (DOCX 1255 kb)

## Abbreviations

ARENA: Alliance for Research in Exercise, Nutrition and Activity based at University of South Australia
BMI: Body mass index
CI: Confidence interval
F: Female participants
ICC: Intra-class correlation coefficient
kg: Kilograms
M: Male participants
m: Metres
MARCA: Multimedia Activity Recall for Children and Adolescents database
MD: Mean difference
Mean: Population mean
mm: Millimetres
Mnth: Months
Non-symp: The non-symptomatic foot
RCT: Randomised controlled trial
SD: Standard deviation of the mean
Symp: The symptomatic foot
VAS: Visual analogue scale
