# Supplementary material for: Custom foot orthoses improve first-step pain in individuals with unilateral plantar fasciopathy: a pragmatic randomised controlled trial
Source: BMC Musculoskelet Disord. 2018 Jul 18;19:222. doi: 10.1186/s12891-018-2131-6 (PMC6052580; doi:10.1186/s12891-018-2131-6)
Supplement: Supplementary file 1 — Instructional. The guidelines for the manufacture and/or design of each intervention. (DOCX 1255 kb) [file 12891_2018_2131_MOESM1_ESM.docx]

**Supplementary File 1 – Overview of Trial Interventions**

Sham intervention (Control Group)

The control group was allocated their existing shoes with a sham insole (0.7 mm cambrelle) as a sham treatment. This was adhered to the existing insole so that no modifications were made to the existing shoe environment.

Shoe Intervention (Shod Group)

The shoe group was allocated a pair of neutral athletic shoes (ASICS Nimbus 14, ASICS Corp, JAPAN, see Figure 1 and shoe specifications in Table 1) with the sham insole (0.7 mm cambrelle) glued to the midsole. When custom foot orthotics are prescribed in clinical practice, they are fitted inside shoes. The use of a new footwear only group in this trial provided the ability to define a positive control group, whereby the effect of the new shoes provided in the orthotic group is compensated for. This allows any differences between the shoe and orthotic group to be a direct result of the orthotic prescribed.


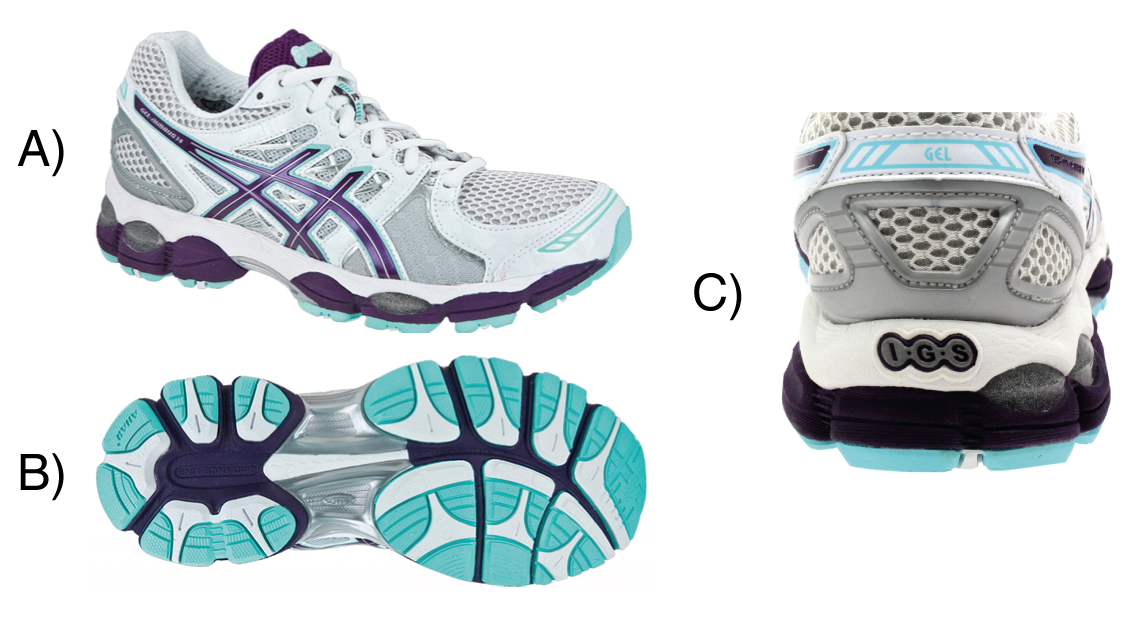


**Figure 1 –** Footwear used in the trial

ASICS Nimbus 14. A) lateral shoe profile, B) sole profile and C) posterior profile showing external heel counter.

**Table 1 –** Technical specifications of ASICS Nimbus 14 athletic shoe.

| **Shoe feature** | | **Technical details** |
| --- | --- | --- |
| Material | *Upper* | Leather and mesh |
|  | *Midsole* | Men’s - Solyte (Density = 53 Shore A)  Women’s - Solyte (Density = 48 / 53 Shore A) |
|  | *Outsole* | Blown rubber (Density = 63 Shore A) |
| Heel-toe gradient | *Men’s* | 10 mm (rearfoot 23mm to forefoot 13 mm) |
|  | *Women’s* | 13 mm (rearfoot 25mm to forefoot 12 mm) |
| Cushioning | *Rearfoot* | GEL |
|  | *Forefoot* | GEL |
| Lasting |  | California (the upper is stitched to a canvas board and directly attached to the midsole) |
| Heel counter |  | External polyurethane (Density = 63 Shore A) |

Custom foot orthotic intervention (orthotic group)

To ensure the custom foot orthotics manufactured were representative of those prescribed in clinical practice, a set of key criteria for orthotic prescription was developed in consultation with a panel of five podiatrists with more than 10 years’ clinical and orthotic prescription experience. This ensured that each device was customised to the needs of the individual. In the development of these criteria, prescription habits of podiatrists were reviewed [1, 2]. These criteria were also based upon a design brief that rather than attempting to restrict and/or control motion, a tissue-stress paradigm was used [3-5]. The overall premise of foot orthotic prescription in this case was to reduce the abnormal load placed on the plantar fascia. The prescription criteria and orthotic prescription for each participant in the orthotic group are provided below.

- The cast of each orthotic will be poured to neutral which is defined as the negative cast, when poured, is held in a vertical position relative to the heel bisection [2].
- The forefoot will be balanced to perpendicular, meaning that the reference platform applied to the plantar forefoot is parallel to the supporting surface and is perpendicular to the rearfoot [2].
- The height of the medial longitudinal arch will be defined by the navicular height in neutral. In addition, minimal plaster is to be added to the midfoot to protect the natural contour of the transverse longitudinal arch (TLA), ensuring the height of the MLA matches the navicular height. We feel this is a more customised approach to arch height prescription and better represents the arch of the foot than the minimal arch fill described by Banwell and colleagues [2]. Further, the navicular is often referred to as the trus of the medial longitudinal arch and is therefore important in investigations where an orthotic is hypothesised to reduce arch deformation.
- An additional measurement will be taken from the base of the 1^st^ metatarsal to the floor in neutral. This provides an additional reference point to define the shape of the medial longitudinal arch.
- A lateral plaster expansion must be added for soft-tissue expansion in weight-bearing.
- No 1^st^ metatarsal cut-outs were to be used. In the Delphi process used by Banwell and colleagues [2], there was concern that stability of the distal device was sacrificed and consensus was not agreed on when to use these modifications. This indicates it is not a common modification prescribed and therefore was not considered.
- Where the plantar fascia visually bowstringed with dorsiflexion of the big toe, a plantar fascia groove will be ground into the device. Grinding into the device rather than adding a plantar fascia accommodation into the plaster work restores the natural transverse longitudinal arch of the foot.
- No Kirby heel skives were to be prescribed given the added pressure they apply to the medial calcaneal tubercle [6].
- All orthotics will have a 350 kg/m^2^ density ethylene-vinyl acetate (EVA) rearfoot post which is balanced to the forefoot [1]. The addition of a rearfoot post provides added medial-lateral stability to the device [7].

**Table 2** in the main manuscript highlights the orthotic prescription provided to each of the 20 participants enrolled into the orthotic group. **Figure 2** below demonstrates the visual appearance of orthotics provided to trial participants.


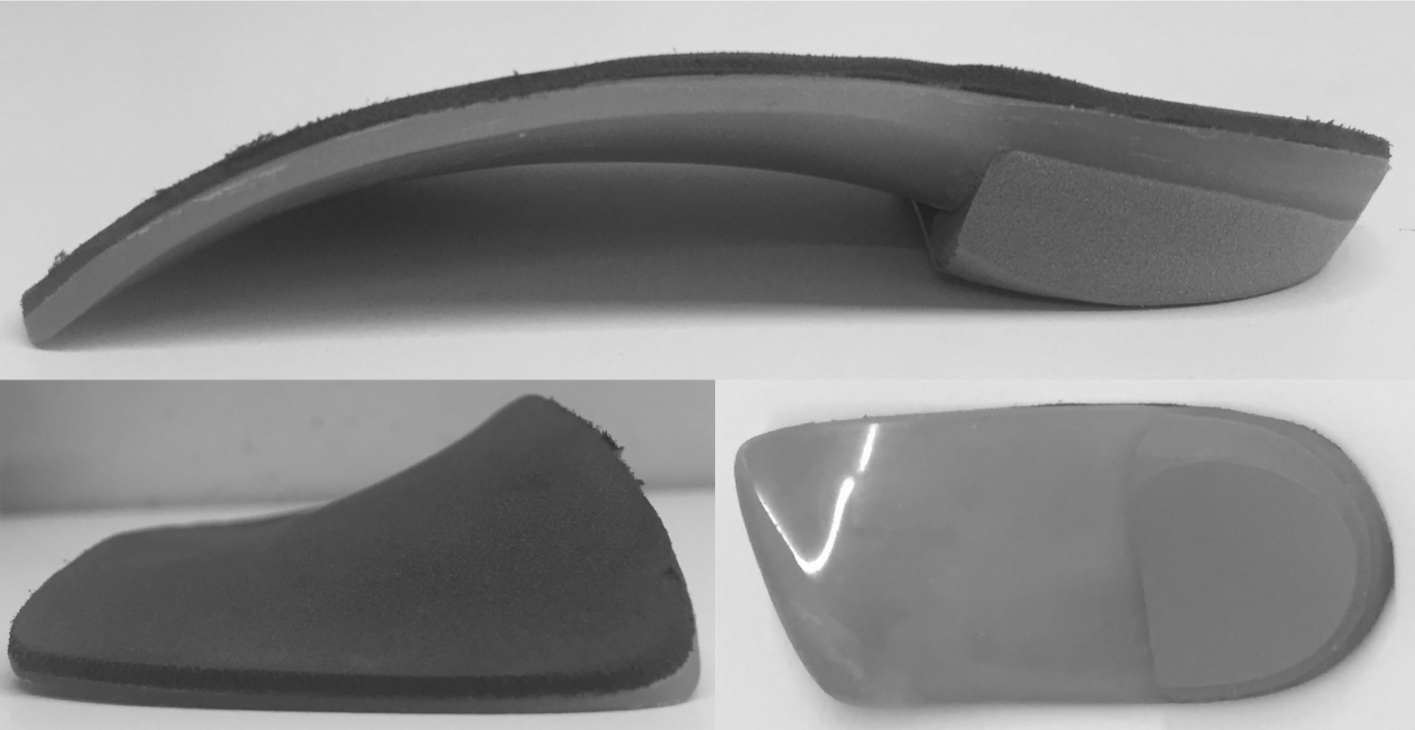


**Figure 2 –** Representative geometric design of the custom foot orthotic provided to a trial participant

**Reference list for supplementary file**

1. Landorf, K., A.-M. Keenan, and R.L. Rushworth, *Foot orthosis prescription habits of Australian and New Zealand podiatric physicians.* Journal of the American Podiatric Medical Association, 2001. **91**(4): p. 174-183.

2. Banwell, H.A., et al., *Consensus-based recommendations of Australian podiatrists for the prescription of foot orthoses for symptomatic flexible pes planus in adults.* Journal of foot and ankle research, 2014. **7**(1): p. 1-13.

3. McPoil, T.G. and G.C. Hunt, *Evaluation and management of foot and ankle disorders: present problems and future directions.* Journal of Orthopaedic & Sports Physical Therapy, 1995. **21**(6): p. 381-388.

4. Payne, C., *The past, present, and future of podiatric biomechanics.* Journal of the American Podiatric Medical Association, 1998. **88**(2): p. 53-63.

5. Mueller, M.J. and K.S. Maluf, *Tissue adaptation to physical stress: a proposed “Physical Stress Theory” to guide physical therapist practice, education, and research.* Physical Therapy, 2002. **82**(4): p. 383-403.

6. Bonanno, D.R., et al., *The effect of different depths of medial heel skive on plantar pressures.* Journal of Foot and Ankle Research, 2011. **4**(Suppl 1): p. O10.

7. Paton, J.S. and S.K. Spooner, *Effect of extrinsic rearfoot post design on the lateral-to-medial position and velocity of the center of pressure.* Journal of the American Podiatric Medical Association, 2006. **96**(5): p. 383-392.
